# Non-emergent care visits in a turkish tertiary care emergency department after 2008 health policy changes: review and analysis

**DOI:** 10.1186/s13690-022-00787-5

**Published:** 2022-01-17

**Authors:** Cihad Dundar, Seydanur Dal Yaylaoglu

**Affiliations:** Department of Public Health, Faculty of Medicine, Ondokuz Mayis University, Samsun, Turkey

**Keywords:** Abuse of health services, Crowding, Emergency department, Government, Hospital, Integrated care, Non-emergent visit, Policy, Triage, Turkey

## Abstract

**Background:**

The Turkish government liberalized national healthcare policies in 2008 enabling Turkish citizens to seek general care in hospital emergency departments (ED). The number of ED visits has exceeded the total population every year for the last ten years. To explain this phenomenon and to identify trends and risk factors for non-emergent visits, we retrospectively reviewed the ED records of a tertiary hospital and the Turkish Ministry of Health bulletin.

**Methods:**

This retrospective record-based study was conducted at a tertiary hospital in Samsun province of Turkey. A total of 87,528 records of adult patients who visited the ED between January 1 and December 31, 2017, were included in this study. We evaluated the pattern of ED use for non-emergent patients by age, gender, nationality, time of visit, means of arrival, ICD (International Classification of Diseases) diagnostic codes, triage codes, number of repeated and out-of-hours visits. We used the Turkish Ministry of Health statistics bulletins to compare the number of ED visits across the country by year.

**Results:**

The non-emergent visit rate in ED was found 9.9%. The rate of non-emergent ED visits was significantly higher in the 18-44 age group, in the female gender, and in those who arrived at the ED without an ambulance. The number of non-emergent visits was very similar between weekends and weekdays but was significantly higher in working hours on weekdays than out-of-hours (*p*<0.001). The most frequent diagnostic code was “Pain, unspecified” (R52) and the rate of repeat visits was 14.8% of non-emergent ED visits. According to binary logistic analysis, non-emergency visits were associated with 18-44 age group (OR = 2.75), female gender (OR = 1.11) and non-ambulance transportation (OR = 9.86).

**Conclusions:**

Our results showed that the 18-44 age group and female gender seek care in the ED for non-emergent problems more than the other parts of the population. The numbers of ED visits in the last decade continued to increase regardless of population growth. The health policy changes may have facilitated access to rapid physical and laboratory examination but also an exacerbation of the free-rider problem in ED services.

## Background

The use of EDs has increased significantly in developed countries. Overcrowding of emergency services is also at critical levels in Turkey. The yearly rate of emergency department visits per person is 0.43 in the USA [1], 0.39 in England [2], 0.31 in Australia, and 1.31 in Turkey [3]. A majority of this increase is attributed to non-emergent visits, which has negative impacts on the quality of care [4]. Crowding caused by non-emergent patients prevents real emergency patients from receiving care, reduces the capability for emergency services, increases waiting times, the workload on personnel, and expenses [5]. Non-emergent ED visits are typically defined as visits for conditions for which a delay of several hours would not increase the likelihood of an adverse outcome [6]. These visits have been described as “inappropriate ED visits” or “avoidable ED visits”, and various definitions exist, ranging from “assigned triage category” to “self-perceived urgency” [7, 8]. Non-emergent ED visits can be considered as the visits of patients not having any accidental or other injuries, do not require emergency treatment, and can be safely treated in primary health care institutions [9]. Yet, non-emergent care in the emergency department is more costly compared to that provided in other care settings, without any improvement in quality [6].

One of the most significant reforms in Turkey in 2008 was to provide free emergency department visits to everyone regardless of their insurance status. This policy change rapidly increased the average number of ED visits [3]. In Turkey, patients have at present the right to seek emergency care without being referred by a physician. Thanks to the abolition of the gatekeeper system with a new law, the obligation of citizens to make their first visits to family health centers (FHC) or other primary health care institutions was suspended in 2009 [10]. Thus, patients gained the right to go to the doctor of their choice at any level health institution, whenever they wanted. As a result, the necessity of obtaining the approval of any doctor or insurance company for this visit was eliminated. Following this new regulation, the annual number of hospital visits increased from 3.8 per person to 6.1, hospital visits accounted for 76% of all healthcare visits between 2008 and 2010 [11]. Subsequently, Turkey switched to the family medicine system across the country in 2011 to increase the quality of primary health care services. In addition, the Turkish Ministry of Health established the Central Physician Appointment System in 2013. In this system, patients can make their medical appointments via 182 Call Center, internet, or mobile application [12]. However, waiting time for the appointments of medical and laboratory examinations was prolonged. As in many countries, emergency and non-emergency care services in Turkey are under the intense pressure of increasing patient numbers. Since access to physicians, radiological and biochemical diagnostic tests are provided faster and free of charge in emergency care services, ED visit rates are still high in Turkey. In an international study covering 34 countries, a shorter waiting time in the emergency room was determined as the third most common reason for going to the emergency room. In most countries, this is unlikely and the percentages are often under 5%. However, only two countries had relatively high percentages: Turkey (20%) and Finland (18%) [13]. The Turkish government liberalized its healthcare policies in 2008, enabling Turkish citizens to seek general care in hospital emergency departments. The number of emergency department visits now exceeds the yearly population for ten years. To explain this phenomenon, we retrospectively reviewed ED visit records in the Turkish Ministry of Health Bulletins published between 2010 and 2017 to evaluate the number of total emergency department visits yearly in Turkey [11, 14–17]. Following the introduction of new health policy regulations, a nonproportional increase of ED visits (36.6%) compared to the increase in-country population (9.6%) was observed during the last decade (Table 1). In 2017, the number of visits to the emergency department of public hospitals in Turkey rose to 101.5 million [14].

**Table 1:** The number of ED visits related to country population in Turkey between 2010-2017 [15, 16]

Year	Number of ED visit	Country Population	ED visit / Population
2010	74.248.061	73.722.988	1,01
2011	75.693.244	74.724.269	1,01
2012	77.156.449	75.627.384	1,02
2013	82.308.086	76.667.864	1,07
2014	84.870.255	77.695.904	1,09
2015	89.457.862	78.741.053	1,14
2016	96.687.756	79.814.871	1,21
2017	101.445.329	80.810.525	1,26

WHO recommends establishing an integrated care organization with a coherent set of methods and models on the funding, administrative, organizational, service delivery, and clinical levels designed to create connectivity, alignment, and collaboration for more effective and efficient health care delivery [17]. These methods and models aim to enhance the quality of care and quality of life, consumer satisfaction, and system efficiency by cutting across multiple services, providers, and settings. Unfortunately, the integrated care system is not available in Turkey. The differences in finance and management are the main obstacles to integration between public, private, and university hospitals and primary health care institutions. The removal of the referral chain caused to break in the communication between primary and secondary health care services, which is one of the essential elements of integration.

In this study, we aimed (a) to determine the characteristics and frequency of non-emergent ED visits in a tertiary hospital, (b) to identify underlying risk factors, and (c) to discuss the relationship between the burden of emergency care and health policy changes in Turkey.

## Methods

This retrospective, a hospital-based study was conducted at the Health Practice and Research Center (HPRC) of Ondokuz Mayis University in Samsun, Turkey. HPRC is modern and a large tertiary referral hospital with 1037 beds and it is about 20 km from the city center. Samsun is the largest city of northern Turkey located on the coast of the Black Sea with a population of 1,312,990 [11]. There are a total of 11 hospitals with emergency services accessible 24/7 in Samsun city center, five of which are state hospitals and six of which are private hospitals. Besides the demographic structure of the society, the health statistics of this province are close to the Turkey average [11]. Therefore, it can be considered that the health services in Samsun are qualitatively similar to those offered in other regions in Turkey.

### Data collection

The records of each patient over 18 years of age who visit the ED of HPRC between January 1, 2017, and December 31, 2017, were included in this study (*n*=87,528). After the ethics committee approval, the hospital’s IT office separated the records of patients presented to the emergency department from the total records in 2017. After the office anonymized the patient’s records by deleting their ID numbers and names, they handed the data to the researchers. Data extraction and pre-analysis were performed between March 1, 2018, and June 15, 2018. The study was approved by the Clinical Research Ethics Committee of Ondokuz Mayis University (CREC: 1500/2018).

### Definition of non-emergent visit

In all hospitals in Turkey, patients undergo effective triage performed by a physician. In the triage system, patients are assigned to a triage code; green, yellow, or red, from the lowest level of emergency to the highest, respectively. The green triage code distinguishes non-emergent patients from others. These are low-risk patients who do not require immediate intervention. They can be treated in primary care or outpatient clinics. According to a circular issued by the Republic of Turkey Ministry of Health, patients having no life-threatening disorder and are stable in general condition after ED examination and who can wait for 1-4 h to be seen in a waiting room are defined as “green zone patients”. While universal emergency care rules apply for patients coded as yellow and red; green-zone patients are told to visit an outpatient clinic or their family physician for assessments, receiving information, and symptomatic treatment, if needed. All visits recorded as green zone patients were considered “non-emergent ED patients” and were included in the study.

### Outcome measures

We evaluated the pattern of ED use for non-emergent patients by age, gender, nationality, time of visit, means of arrival, ICD diagnostic codes, and triage codes. We also compared the frequency of out-of-hours ED visits in emergent and non-emergent patients, as problems with access to primary care and outpatient clinics may have affected ED visits. The traditional Turkish business hours are 8:00 a.m. to 5:00 p.m., Monday to Friday, including healthcare institutions. For this reason, we defined these hours as “working hours”. Means of arrival were simply categorized into two groups: Ambulance, and non-ambulance. Non-ambulance includes all other means of ambulatory or non-medical emergency transport (any mechanized means other than an ambulance). All refugees who present to ED of HPRC were gathered under the heading “Foreigner”. There are officially around 22,000 Iraqis, 6,000 Syrians, and 1,500 Afghans in Samsun.

### Statistical analysis

Data were analyzed using the SPSS 22.0 software program (SPSS Inc., Chicago, IL, USA). Proportional differences between groups for each variable were compared using the Chi-square test. The value of *p*<0.05 was considered statistically significant. Binary logistic regression analysis was used to define the impact of individual risk factors on non-emergent visits. The value of *p*<0.05 was considered statistically significant.

## Results

For the year 2017, 78,903 (90.1%) of the 87,528 visits to ED of HPRC were emergent, while 8,625 (9.9%) were non-emergent. The median age of the non-emergent patients was 30 years (range: 18-97) and 39 years in the emergent patient group (range: 18-100). As shown in Table 2, the rate of non-emergent visits was significantly higher in the 18-44 age group, and in the female gender. Non-emergent patients tended not to use ambulance vehicles. The number of non-emergent visits was very similar between weekends and weekdays but was significantly higher in working hours (08:00 am – 05:00 pm) than out-of-hours on weekdays (*p*<0.001).

**Table 2:** Some characteristics of ED visitors by the level of emergency

Characteristics	Non-emergen n (%)	Emergent n (%)	Total	p
Gender				
Male Female	4110 (47.7) 4515 (52.3)	36522 (46.3) 42381 (53.7)	40632 46896	**0.015**
Nationality				
Turkish Foreign	8516 (98.7) 109 (1.3)	78032 ((98.9) 871 (1.1)	86548 980	0.18
Age groups (year)				
18-44 45-64 >65	6352 (73.6) 1655 (19.2) 618 (7.2)	46586 (59.0) 19342 (24.5) 12975 (16.5)	52938 20997 13593	**<0.001**
Mode of arrival				
Ambulance Non-ambulance	40 (0.5) 8585 (99.5)	3848 (4.9) 75055 (95.1)	3888 83640	**<0.001**
Visit day				
Weekdays Weekends	6250 (72.5) 2375 (27.5)	57341 (72.7) 21562 (27.3)	63591 23937	0.690
Visit hours on weekdays				
Working hours Out-of-hours	3145 (50.3) 3105 (49.7)	27379 (47.7) 29962 (52.3)	30524 33067	**<0.001**
Total	8625 (100.0)	78903 (100.0)	87528	

As a result of logistic regression analysis, 18-44 age (OR = 0.98; CI = 0.978-0.980), female gender (OR = 1.12; CI = 1.073-1.174) and non-ambulance transportation (OR = 9.75; CI = 7129-13.324) were all found to be related with non-emergent ED visit statistically significant (*p*<0.001).

The most frequent non-emergent visits occurred in autumn and on Mondays, Wednesdays, and Tuesdays. While weekday and seasonal distributions were similar in emergent and non-emergent visits, there were differences according to monthly periods.

When the initial visits of the patients who had repeated non-emergent ED visits during the year were excluded from the evaluation, 1275 repeated ED visits were identified, which accounted for 14.8% of all green zone visits.

Some patients had more than one diagnostic code, as 10,037 diagnostic codes were observed in 8,625 non-emergent ED visits. The most frequent diagnostic code was “Pain, unspecified” (R52) (Table 3).

**Table 3:** Distribution of ICD codes of patients who presented to the ED

	Non-emergent	Emergent
**ICD code**	**n (%)**	**n (%)**
R52. Pain, unspecified	3356 (33.4)	24,062 (30.4)
J39.9. Disease of upper respiratory tract, unspecified	1865 (18.7)	7409 (9.3)
M79. Other and unspecified soft tissue disorders, not elsewhere classified	281 (2.8)	1602 (2.0)
R10.9. Unspecified abdominal pain	254 (2.5)	2130 (2.7)
R07.0. Pain in throat	234 (2.3)	1149 (1.5)
R05. Cough	217 (2.2)	1900 (1.4)
R51. Headache	208 (2.1)	3860 (4.9)
R11. Nausea and vomiting	175 (1.7)	1998 (5.1)
H10. Conjunctivitis	133 (1.3)	268 (0.3)
M54.5. Low back pain	112 (1.1)	1362 (1.7)
Other	3202 (31.9)	33,163 (42.0)

## Discussion

The rate of non-emergent ED visits (9.9%) was lower than those reported in previous studies which are between 4.8% and 90% [4, 18, 19]. Although the rates of ED use vary by country and region due to these different definitions, some factors such as socioeconomic conditions, hospital admission policy, private health insurance coverage, personal health system utilization, and chronic disease profile of the population have been shown to drive geographical variation in ED visits [4, 20, 21]. The most challenging part of these studies was determining non-emergent ED visits, which were largely estimated, relying on diagnostic criteria or the judgment of clinical staff in previous studies [21].

According to the Emergency Health Services Regulation published by the Ministry of Health of the Republic of Turkey in 2000, hospital emergency departments are the units that provide emergency health services within the body of secondary and tertiary public and private health institutions and organizations. These services and units are commissioned for providing emergency medical care to the emergency patients and injured people who apply directly to them or are brought by the teams affiliated to the provincial ambulance service department. They are also responsible for keeping records of the service provided. All public and private hospitals must provide the first medical intervention and medical care by accepting emergency patients and injured people 24 h a day [22]. Although there are eleven hospitals in Samsun city center, hosting a big part of the population within the province, the number of emergency department visits in HPRC, which is 20 km away from the center, was found to be still quite high. In the same year, the number of ED visits was 504,708 in a tertiary level state hospital with 1140 beds in Samsun city center. The city of Manisa located in Western Turkey and Kahramanmaraş located in Eastern Turkey have similar population numbers and socioeconomic status with Samsun. In 2017, the number of patients presented to the ED was 347,626 in a state hospital with 415 beds in Manisa and 677,720 in a state hospital with 1040 beds in Kahramanmaraş. The ratios of ED admissions to the total number of admissions in state hospitals were 28.4%, 31.7%, and 24.6% in Samsun, Manisa, and Kahramanmaraş, respectively [14]. In 2017, the ED visits per person were 1.7 in Samsun, 1.3 in Manisa, and 1.3 in Kahramanmaraş, parallel to the average of Turkey [11, 14]. There are no incentives for the care layers (primary-secondary and tertiary) to work together in Turkey. A performance-based remuneration system for hospital personnel has also created competition between the levels of care [22]. Care coordination is a foundational element of an effective and efficient integrated care delivery system involved in a patient’s care to facilitate the appropriate delivery of health care services [23]. The essential element of integrated care and transition between the care layers is reinforced primary care, particularly critical for patients with complex or urgent needs [17]. Therefore, effective care requires establishing a relationship between primary care health personnel and key specialists, hospitals, and community-based organizations.

In the literature, similar to our study, analysis of the demographic characteristics predictive of non-emergent patients showed that age, sex, and arrival to the ED were found to be related to non-emergent ED visits [9, 18, 19]. The fact that non-emergent patients were younger than emergent patients and were significantly more in the 18-44 age group was consistent with other studies in the literature [4, 8, 18, 24]. The studies conducted in France and Taiwan stated that the average ages of patients who visited the emergency department for non-emergent reasons were 36 and 37 years, respectively, and about three-quarters of them did not have a chronic disease [25, 26]. Based on these results, it can be concluded that non-emergent visits are mostly made by young and middle-aged adults. The non-emergent visit rate was significantly higher in patients arriving by their means than patients who were brought by ambulances. This difference may be because ambulance patients are pre-evaluated before arrival. Whether the patient’s medical condition is an emergency or not is evaluated both at initial request to the emergency call center and when the emergency ambulance team reaches the patient. Therefore, the preliminary assessment made by the 112 Emergency Call Center and ambulance crew seems to play an important role as a gatekeeper to prevent non-emergent ED visits. While patients do not pay any expense for diagnosis or treatment in emergent or very-emergent triage codes in the ED, they do pay a contribution fee for non-emergent visits. However, it is far from being a deterrent because the amount of the contribution payment taken from green zone patients is very low (3-5 US dollars) [27]. Otherwise, all primary care services are provided free of charge.

Some publications state that EDs are preferred even with costs concerns, this was not the case for our study. The fact that the rate of recurrent admissions in patients with previous non-emergent visits was 14.8% also supports this theory. In addition, the data was not obtained from a national database, and it is unknown whether patients visited different EDs within the same year. Our study determined that there were patients with 13, 16, even 20 non-emergent visits.

The high number of emergent and non-emergent visits in the first days of the week may be related to the tendency to go to the ED of patients who cannot get an outpatient appointment immediately after the weekend. As in another study conducted in Turkey, non-emergent visits were found significantly higher during business hours [12]. The most common diagnostic codes recorded in non-emergent visits were pain, upper respiratory tract infection, soft tissue disorder affecting the person’s quality of life. This kind of non-emergent visit may be related to quick ED assessment without waiting queues, yield rapid test results, and promptly provided service. Studies conducted using surveys or hospital records also support this theory [4, 8, 12, 28, 29]. In Samsun, the population number per physician of FHC was above the average for Turkey (Turkey average 3124 vs. Samsun 3266) [11]. In the year succeeding this study’s data collection, the Ministry of Health of Turkey initiated an out-of-hours service in FHC to decrease non-emergent visits to EDs and outpatient clinics. In many studies, it was found that reasonable access to primary care providers such as GPs and continuity of care measured by seeing the same family doctor were essential factors in reducing non-emergent ED visits [30, 31]. In the current health system, while it may seem that patients being unable to access immediate health service leads them to prefer EDs [29], many studies have shown that the level of service, working hours, and increased professionalism do not decrease the use of EDs [8, 24, 32–34].

Our study has some limitations. Due to the record-based nature of our research, we could not obtain some basic data to elucidate cause-effect relationships. The records of patients who visited the ED did not include some critical data such as distance to hospital, number of visits, education level, and marital status. Even our data was collected from a large district hospital, usage of a single hospital archive might limit the generalizability of our findings. Another limitation is the uncertainty of whether there were errors in coding the patients. To detect non-emergent ED visits, we used patient records coded as “green-zone patients” in the hospital records. Since the data used were anonymized, and we did not know about the prognosis of the patients, we could not check whether coding errors might be in the records.

## Conclusions

Our results showed that the 18-44 age group, and female gender seek care in the ED for non-emergent problems more than the other parts of the population. The numbers of ED visits in the last decade have continued to increase regardless of population growth throughout the country. The health policy changes may have facilitated access to rapid physical and laboratory examination but also an exacerbation of the free-rider problem in ED services.

To reduce the non-emergent ED visits, further studies on the development of triage criteria, and the impact of the legal changes on the emergency department are required. Besides the health care needs of the populations, reasons. why FHC and outpatient clinics are not preferred by non-emergent patients should also be further investigated.

## Data Availability

The data that support the findings of this study are available on request from the corresponding author, [CD]. The data are not publicly available due to restrictions their containing information that could compromise the privacy of research participants.

## Abbreviations

ED: Emergency department
FHC: Family Health center
HPRC: Health Practice and Research Center
ICD: International Classification of Diseases

## Declarations

(KAEK-1500). Written informed consent was not obtained from the patients since the study was conducted on anonymous data of hospital records.
